# Artificial Mini Dendritic Cells Boost T Cell–Based Immunotherapy for Ovarian Cancer

**DOI:** 10.1002/advs.201903301

**Published:** 2020-02-05

**Authors:** Shanshan Cheng, Cong Xu, Yue Jin, Yu Li, Cheng Zhong, Jun Ma, Jiani Yang, Nan Zhang, Yuan Li, Chao Wang, Zhiyou Yang, Yu Wang

**Affiliations:** ^1^ Department of Obstetrics and Gynecology Shanghai Key Laboratory of Gynecologic Oncology Renji Hospital School of Medicine Shanghai Jiaotong University Shanghai 200127 P. R. China

**Keywords:** dendritic cells, immunotherapy, nanovaccines, ovarian cancer, T cells

## Abstract

Ovarian cancer is the most lethal gynecological malignancy with high recurrence rates and low survival rates, remaining a disease of high unmet need. Cancer immunotherapy, which harnesses the potential of the immune system to attack tumors, has emerged as one of the most promising treatment options in recent years. As an important form of immunotherapy, dendritic cell (DC)–based vaccines have demonstrated the ability to induce an immune response, while clinical efficacy of DC vaccines remains unsubstantiated as long‐term benefit is only reported in a restricted proportion of patients. Here, a biomimetic nanovaccine derived from DCs is developed through cell membrane coating nanotechnology. This nanovaccine, denoted “mini DC,” inherits the ability of antigen presentation and T cells' stimulation from DCs and is shown to elicit enhanced activation of T cells both in vitro and in vivo. In a mouse model of ovarian cancer, mini DCs exhibit superior therapeutic and prophylactic efficacy against cancer including delayed tumor growth and reduced tumor metastasis compared with DC vaccine. These findings suggest that mini DCs may serve as a facile and potent vaccine to boost anticancer immunotherapy.

## Graphical abstract

This work reports a biomimetic nanovaccine derived from dendritic cells (DCs). This nanovaccine possesses abilities of antigen presentation and T cells' stimulation and is shown to elicit enhanced activation of T cells both in vitro and in vivo. It also exhibits superior therapeutic and prophylactic efficacy against tumors compared with DC vaccine in a mouse model of ovarian cancer.

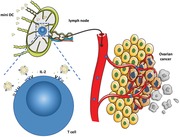

## 1 Introduction

Ovarian cancer is the fifth leading cause of cancer‐related deaths in women and the deadliest gynecologic malignancy, with over 22 000 new cases and 14 000 deaths due to the disease estimated in the United States in 2018.^1^, ^2^ The 10 year overall survival rate remains <30% over the past 30 years due to the fact that the vast majority (more than 70%) of patients in clinic are diagnosed at advanced stages (stage III or stage IV) of disease with widespread dissemination in pelvic and abdominal cavity.^3^, ^4^ The current first‐line treatment for ovarian cancer is debulking surgery combined with paclitaxel‐ and carboplatin‐based chemotherapy.^5^, ^6^ Although many patients display a good initial response to these conventional therapies, most of them experience relapse and ultimately develop platinum resistance within 6 to 18 months.^7^, ^8^ Hence, there is a compelling need to explore novel and effective therapies for the management of ovarian cancer.

With recent breakthroughs in several areas of immunotherapy, it has attracted significant attention of gynecologists.^9^ Substantial evidence suggested that ovarian cancer patients could benefit from immunotherapy.^10^, ^11^ As an important form of immunotherapy, dendritic cell (DC)–based cancer vaccines have gained notable advances in recent years with one of them being approved by the US Food and Drug Administration (FDA) for the treatment of prostate cancer.^12^, ^13^ To date, DCs have been mostly used after being pulsed ex vivo with tumor‐associated antigens (TAAs) or whole tumor cell lysates. These primed autologous DCs are anticipated to activate naïve and or induce memory tumor‐specific T cells when they migrate from the administration site to the draining lymph nodes (dLNs). Both preclinical and clinical data reveal that DC vaccination could induce effective antitumor immunity in vivo. However, only a limited number of patients benefited from DC vaccination in clinical trials conducted during past two decades.^14^, ^15^ In a recently reported phase I study of DC vaccination in patients with recurrent ovarian cancer, only half of evaluable patients exhibited detectable T‐cell response, although those patients with vaccine responsiveness benefited from significantly longer progression‐free survival compared with those who failed to respond to DC vaccination.^16^, ^17^ Several mechanisms, including restricted migration of DCs to draining lymph nodes, downregulation of TAAs and major histocompatibility complex (MHC) on tumor cells, immunosuppressive tumor‐associated microenvironment (TAM), and metabolic constraints on the activation of tumor‐associated DCs, may cause the limited clinical efficacy of DC vaccines.^18^, ^19^, ^20^, ^21^, ^22^ In addition, for now there is no consensus on the standardization of DC vaccine manufacture, which may have significant influence on the viability of DCs and potency of vaccine.^15^ Therefore, alternative strategies are eagerly needed to improve the performance of DC‐based immunotherapy.

More recently, cell membrane coating nanotechnology has gained much attention as it offers a feasible way to modify nanoparticles with natural cell membranes.^23^, ^24^, ^25^ Through a simple process of extrusion, cell membranes can be readily fused onto synthetic polymeric cores. These nanoparticles obtain some unique properties of donor cells, enabling them to exert donor cells' function without limitations of cellular structure, size, and viability.^26^ One of the most promising examples is the nanoparticles coated with erythrocyte membranes (RBC‐NPs), acting as decoys to absorb pathological pore‐forming toxins and detain auto‐antibodies.^27^

**Figure 1:**
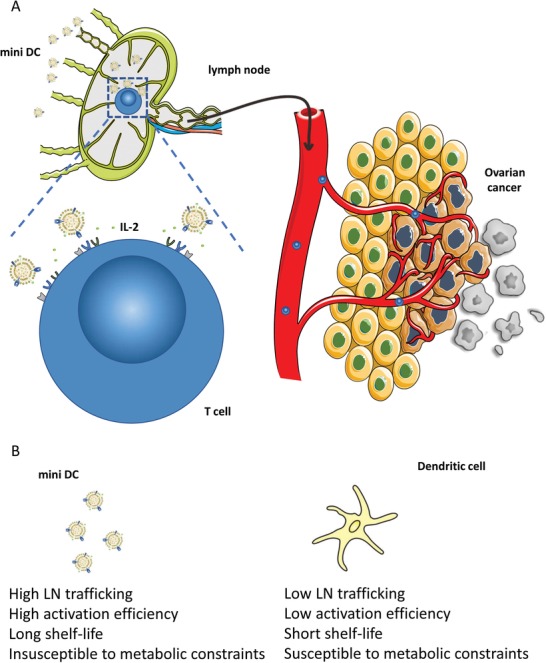
A) Schematic representation of the mechanism by which biomimetic mini DC nanovaccine for enhanced adaptive T cell–based immunotherapy for ovarian cancer. B) Comparison between mini DC and conventional DC cells.

Inspired by the aforementioned biomimetic method, we have developed dendritic cell–like nanoparticles (denoted “mini DC”) through coating cell membranes extracted from ovarian cancer cell lysate‐primed DCs onto interleukin‐2 (IL‐2)‐loaded biodegradable poly(lactic‐*co*‐glycolic acid) (PLGA) nanoparticles using an extrusion approach.^28^, ^29^ By presenting DCs' functional plasma membrane proteins (such as MHC, CD86, and CD40) on its surface, mini DC is expected to mimic DCs' antigen presentation ability and releases IL‐2 in a paracrine manner, thus activating T cells and provoking robust antitumor immune response without being affected by immunosuppressive TAM and physiological barrier during migration and antigen presentation (**Figure** **1**).^30^, ^31^ Furthermore, the feasible storage condition and long shelf‐life offer better clinical maneuverability compared with DC vaccines. Here we demonstrate that the administration of nanoparticulated mini DC can induce systemic immune responses through its specific interaction with T cells and efficiently inhibit growth and metastasis of ovarian cancer, suggesting its potential as a robust and safe strategy for cancer immunotherapy.

## 2 Results and Discussion

### 2.1 Preparation and Characterization of Mini DC

**Figure 2:**
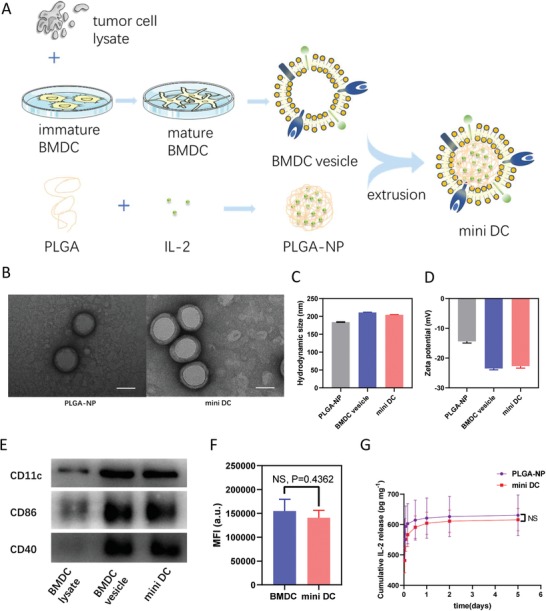
Preparation and characterization of mini DC. A) Schematic illustration for the preparation of mini DC, which were fabricated by coating IL‐2 loaded PLGA‐NP with tumor cell lysate‐primed DC membrane. B) Representative TEM images of PLGA‐NP (left) and mini DC (right) stained with uranyl acetate. Scale bar: 100 nm. C) Hydrodynamic size and D) zeta potential of PLGA‐NP, BMDC vesicle, and mini DC measured by dynamic light scattering. E) Membrane‐specific protein markers of DC lysate, BMDC vesicle, and mini DC characterized by western blotting. F) Fluorescence intensity of BMDC (≈2 × 10^6^ suspended in 100 µL PBS) and mini DC (0.25 mg mL^−1^ protein content and 100 µL of suspension) stained with PE‐labeled anti‐mouse MHC II antibody. G) Release kinetics of IL‐2 of PLGA‐NP and mini DC in PBS at 37 °C measured by ELISA. G,H) Data are means ± standard deviation (SD) (*n* = 3). Significance was assessed using unpaired two‐tailed *t*‐test. NS: no significance.

To fabricate mini DC, bone marrow–derived dendritic cells (BMDCs) were pulsed with homogenized ID8 murine ovarian tumor cell lysate after HOCl oxidation as reported in previous studies.^32^, ^33^ Trypan blue staining showed that HOCl‐oxidized tumor cells had a cell viability of 0%, and homogenization could effectively enhance the uptake of tumor cell lysate by BMDC (Figure S1, Supporting Information). Then the membranes of primed BMDC were isolated and extruded with PLGA polymeric cores, which were synthesized using a double emulsion method (**Figure** **2**A).^28^ As one of important cytokines involving in the expansion and differentiation of T cells, IL‐2 was loaded into the PLGA nanoparticle (PLGA‐NP) during the synthesis process. Transmission electron microscopy (TEM) imaging after uranyl acetate negative staining showed that PLGA‐NP was fully encapsulated into DCs' membrane and the resulting nanoparticles, mini DC, possessed a core–shell structure with a diameter of about 160–170 nm (Figure 2B; Figure S2, Supporting Information). Dynamic light scattering also revealed that mini DC gained an increase of ≈20 nm, which is consistent with the thickness of double layers of cell membrane, in hydrodynamic diameter compared with the bare PLGA‐NP (Figure 2C; Figure S3A, Supporting Information).^34^ The surface zeta potential of mini DC decreased from −14 to −22 mV, similar to that of DCs' membrane‐derived vesicle (BMDC vesicle), also indicating that the PLGA‐NP had been wrapped in natural BMDC membranes successfully (Figure 2D; Figure S3B, Supporting Information). The retaining of critical DCs' membrane proteins, including CD11c, CD86, and CD40, on the mini DC surface was further confirmed by immunoblotting and Coomassie blue staining (Figure 2E; Figure S4, Supporting Information). To verify the right‐side‐out orientation of these proteins, immunostaining of MHC II was conducted with phycoerythrin (PE)‐labeled anti‐MHC II antibody. As a result, the fluorescence intensity of mini DC was comparable to that of DCs with an equal amount of surface proteins, further proving that mini DC presented cell membrane in the same orientation as their source cells (Figure 2F).^35^ As for IL‐2 release in phosphate‐buffered saline (PBS) at 37 °C, both PLGA‐NP and mini DC displayed a burst release during the first 12 h and similar total release in 5 days (Figure 2G). Additionally, once lyophilized and preserved at −20 °C, mini DC showed good stability as the protein content remained over 80% within 4 weeks and over 65% within 8 weeks, which also demonstrated its relatively less strict storage condition (Figure S5A, Supporting Information). Overall, a successful translocation of DCs' membrane onto PLGA‐NP and a stable biomimetic structure of mini DC were affirmed, ensuring the feasibility of subsequent experiments. Of special note, possessing an average diameter of <200 nm might also facilitate its free trafficking to the lymph nodes (Figure S5B, Supporting Information).^36^, ^37^

### 2.2 In Vitro Specific Binding and Activation of T Cells by Mini DC

**Figure 3:**
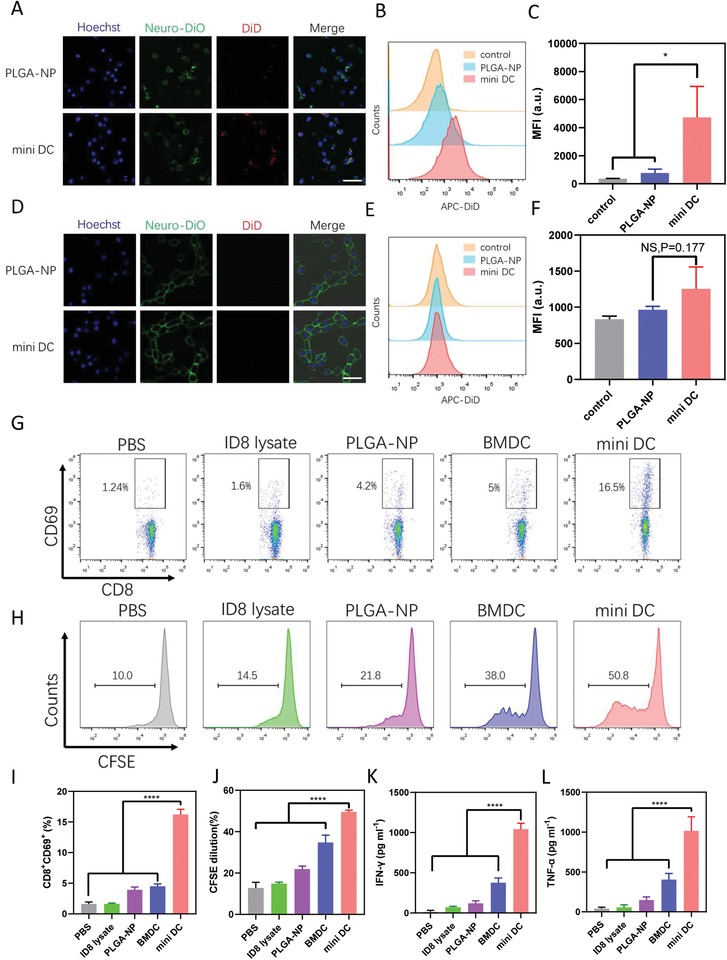
Interaction between mini DC and T cells in vitro. A) CLSM images of primary CD8^+^ T lymphocytes and D) NIH‐3T3 cells after incubation with PLGA‐NP or mini DC at 4 °C for 1 h. Blue represents nuclei and red represents nanoparticles. Scale bar: 50 µm. Representative flow cytometry histogram and MFI quantifications of B,C) T cells and E,F) NIH‐3T3 cells incubated with different nanoparticles. G,I) Flow cytometric analysis and percentage of CD8^+^CD69^+^ T cells after incubation with various formulations for 24 h. H,J) Flow cytometric analysis of CFSE‐labeled primary CD8^+^ T‐cell proliferation cultured with different formulations for 3 days. CFSE dilution was used for quantification of T‐cell proliferation. K,L) Concentration of IFN‐γ and TNF‐α in the cell culture supernatant of T cells after 3‐day stimulation with mini DC and other formulations. For panels (C), (F), and (I)–(L), data were represented as mean ± SD. *n* = 3 for panels (C) and (F) and *n* = 4 for panels (I)–(L). Statistical analysis was performed using C,F) unpaired two‐tailed Student's *t*‐test and I–L) one‐way ANOVA with Dunnett's posthoc analysis. ****p < 0.0001 and *p < 0.05. NS: no significance.

The interaction between the resultant biomimetic mini DCs and T cells is the key to exert its immune stimulation function. Mini DC synthesized with 1,1′‐dioctadecyl‐3,3,3′,3′‐tetramethylindodicarbocyanine, 4‐chlorobenzenesulfonate salt (DiD)‐labeled PLGA‐NP was incubated with CD8^+^ T cells isolated from mouse spleens at 4 °C, with PLGA‐NP being tested as controls. After 1 h incubation and washing, confocal laser scanning microscopy (CLSM) images displayed that cells incubated with mini DC showed significant nanoparticle binding compared with those incubated with PLGA‐NP (**Figure** **3**A). Flow cytometry also confirmed that significantly increased mean fluorescence intensity (MFI) was observed on cells incubated with mini DC, but not with PLGA‐NP (Figure 3B,C). In contrast, no significant difference of fluorescence was observed when mini DC and PLGA‐NP were incubated with NIH‐3T3 cells at the same condition (Figure 3D–F). These findings demonstrate the binding ability of mini DC to T cells conferred by their DCs' membrane enclosing. The interaction between DCs' membrane proteins, including MHC and costimulatory molecules, with T‐cell receptor (TCR) and CD28 on T cells may attribute to this specific adhesion.^38^

We then investigated the ability of mini DC in T‐cell activation in vitro. Primary CD8^+^ T cells isolated from mouse spleens were incubated with mini DC at 37 °C, with PBS, ID8 lysate, PLGA‐NP, and BMDC serving as controls. After 1 day incubation, T cells were collected and analyzed with flow cytometry. Mini DC induced threefold higher percentage of CD69^+^‐activated T cells than BMDC (Figure 3G,3). T‐cell proliferation assay, in which carboxyfluorescein succinimidyl ester (CFSE)‐labeled T cells were used, was also conducted to further evaluate the stimulation ability of mini DC. After 3 days incubation, T cells and cell culture supernatants were collected for flow cytometry and enzyme‐linked immunosorbent assay (ELISA). As measured by CFSE dilution, mini DC promoted the highest proliferation of CD8^+^ T cells (Figure 3H,J; Figure S6, Supporting Information). The result of ELISA also indicated that mini DC could strongly promote the secretion of proinflammatory cytokines interferon (IFN)‐γ and tumor necrosis factor (TNF)‐α from T cells, which are important markers of activated cytotoxic T cells (Figure 3K,L).^39^

### 2.3 Elicitation of Robust T‐Cell Response by Mini DC In Vivo

**Figure 4:**
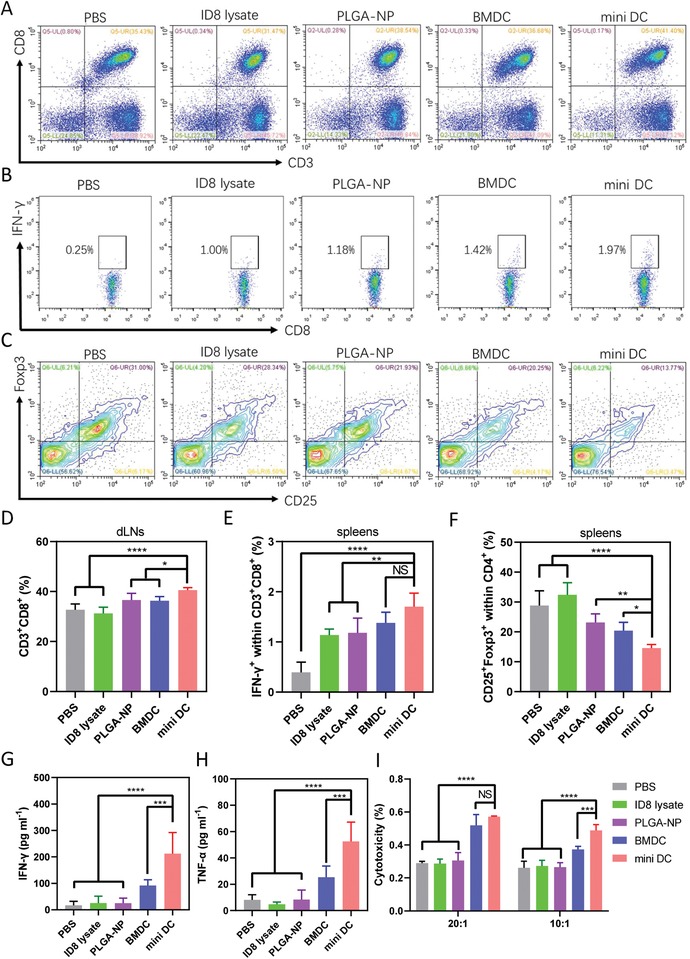
In vivo activation of T cells by mini DC. A) Representative flow cytometry scatter plots and D) frequency of CD3^+^CD8^+^ T cells in dLNs of mice 3 days after immunization with six dosages of PBS, ID8 lysate, PLGA‐NP, BMDC, or mini DC (*n* = 5 biologically independent animals in each group). Flow cytometry analysis and percentage of B,E) IFN‐γ^+^CD8^+^ effector T cells and C,F) Foxp3^+^CD25^+^CD4^+^ regulatory T cells isolated from spleens of mice receiving different vaccinations. G) IFN‐γ and H) TNF‐α levels in serum of immunized mice measured by ELISA. I) Ex vivo cytotoxicity of CD8^+^ T cells isolated from spleens of immunized mice 3 days after vaccination with different vaccine formulations (*n* = 4). CD8^+^ T cells (effector cell) and ID8 cells (target cell) were cocultured at ratios of 20:1 and 10:1 (E:T) for 10 h. In panels (D)–(I), representative data were expressed as mean ± SD. One‐way ANOVA with Dunnett's posthoc analysis was used to calculate statistical significance. ****p < 0.0001, ***p < 0.001, **p < 0.01, and *p < 0.05. NS: no significance.

Encouraged by the T‐cell activation ability of mini DC in vitro, we then explored the immune stimulation and T‐cell activation property of mini DC in vivo. Female C57BL/6 mice were injected subcutaneously at the tail base with 100 µL various formulations of vaccines, including ID8 lysate, PLGA‐NP, equivalent ID8 lysate‐pulsed BMDC, and mini DC twice a week for 3 weeks. Three days after six doses of vaccination, mice were sacrificed, and flow cytometry analysis showed significantly higher percentage of CD3^+^CD8^+^ T cells in dLNs from mice treated with mini DC over other four control groups (**Figure** **4**A,D). Spleens of vaccinated mice were also harvested for flow cytometry analysis, and the result showed that mini DC–immunized mice generated more CD8^+^IFN‐γ^+^ effector T cells (T_eff_) than other groups, although the difference is not statistically significant when compared with the BMDC group (Figure 4B,E). Furthermore, the percentage of CD4^+^CD25^+^Foxp3^+^ regulatory T cells (T_reg_) in mini DC–vaccinated mice was the lowest among all groups and T_eff_ outnumbered T_reg_ by about 6.5‐fold in spleens, which is 1.5 times higher than that of BMDC‐vaccinated mice (Figure 4C,F; Figure S7, Supporting Information). Similar to the result of in vitro study, the IFN‐γ and TNF‐α levels in the serum of mini DC–treated mice increased by 2.3 and 2 times when compared with mice administrated with BMDC.

To further determine whether the adaptive immune response induced by mini DC was tumor specific and the activated T cells possessed antigen‐specific cytotoxicity, we cocultured live ID8 cells (target cell) with CD8^+^ T cells (effector cell) isolated from spleens of immunized mice. Although T cells from BMDC and mini DC–treated mice exhibited similar cytotoxic effect when the effector:target ratio is 20:1, stronger cytotoxicity was observed in T cells from mini DC–vaccinated mice compared with that of BMDC‐vaccinated mice when the ratio decreased to 10:1 (Figure 4). Collectively, these data demonstrated that mini DC could efficiently elicit systemic and tumor‐specific immune response in mice and displayed advantage over BMDCs in potentiating the antitumor effect of T cells.

### 2.4 Inhibition of Tumor Growth after Intervention of Mini DC

**Figure 5:**
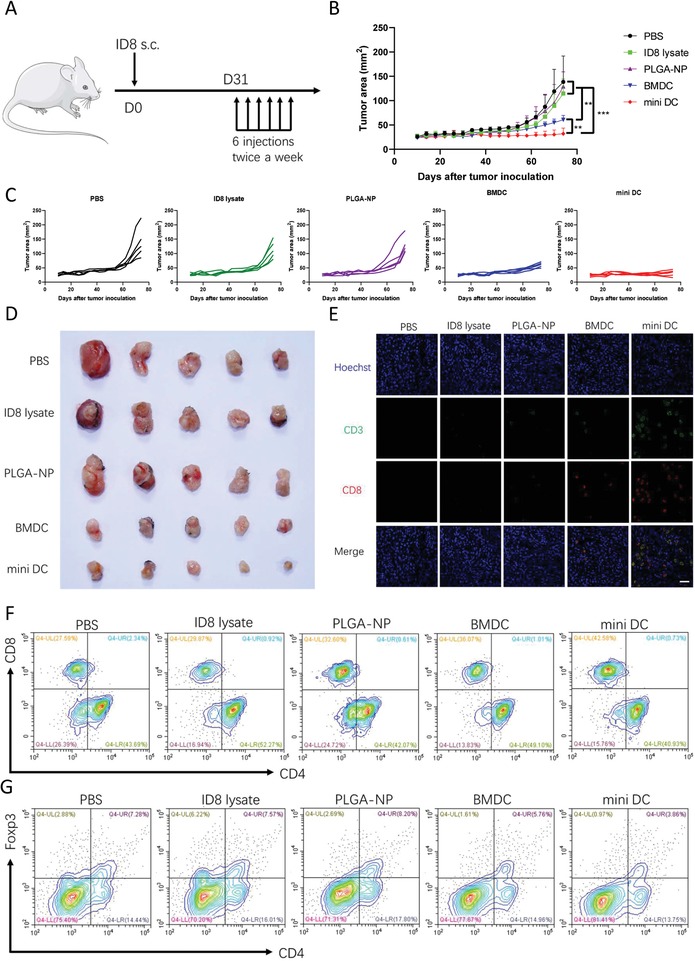
Immune stimulation ability and therapeutic efficacy of mini DC in tumor‐bearing mice. A) Schematic diagram for the therapeutic regimen of mini DC in tumor‐bearing mice. The mice inoculated with ID8 mouse ovarian cancer cells subcutaneous (s.c.) received six doses of various vaccine formulations, and tumor growth was measured over time (*n* = 5 biologically independent animals in each group). B) Average and C) individual tumor growth curves as well as D) the digital tumor mass images of tumor‐bearing mice treated with indicated vaccine regimens. When the tumor area of the control group exceeds 200 mm^2^, mice were sacrificed, and tumors and spleens were collected for section and analysis of the immune cell profile. E) Representative immunofluorescence staining images of tumor sections from different groups. Green represents CD3 and red represents CD8. Scale bar: 50 µm. F) Representative flow cytometry contour plots of CD8^+^CD3^+^ T_eff_ and G) Foxp3^+^CD4^+^ T_reg_ in the spleens of tumor‐bearing mice at the end of the study. Data in panel (B) show mean ± SD and were analyzed by two‐way ANOVA with Tukey's multiple comparisons test. ****p < 0.0001 and **p < 0.01.

The high efficacy of mini DC to activate T cells in vitro and in vivo prompted us to examine whether the induced antitumor immune response can inhibit the growth of tumor in tumor‐bearing mice. The mouse ovarian cancer model was established by injection of ID8 cells into the right flank of female C57BL/6 mouse subcutaneously. One month after tumor inoculation, total six doses of different vaccine formulations were administrated subcutaneously at the tail base with a twice‐per‐week frequency (**Figure** **5**A). ID8 lysate or PLGA‐NP did not offer any significant tumor growth inhibition compared with PBS control. In contrast, BMDC and mini DC vaccine were found to delay the tumor growth after vaccination while mini DC manifested the maximum therapeutic efficacy at end of the monitoring when the tumor area of PBS control group reached 200 mm^2^ (Figure 5B–D).

To find out the mechanism underlying the enhanced antitumor effect of mini DC, we collected the tumor masses for immunofluorescence staining and terminal deoxynucleotidyl transferase 2′‐deoxyuridine 5′‐triphosphate nick end labeling (TUNEL) staining. Immunofluorescence staining images showed that there were more CD8^+^ cytotoxic T lymphocytes (CTLs) present in the tumors of mini DC–vaccinated mice than other groups (Figure 5E). The increased intratumor CTLs infiltration might contribute to marked apoptosis of tumor cells as illustrated in TUNEL staining images (Figure S8, Supporting Information).^40^ This was in agreement with the finding that tumor‐infiltrating CD8^+^ T cell was of prognostic value for survival of human ovarian cancer patients.^10^ Consistently, the frequency of CD3^+^CD8^+^ effector T cells in the spleens of mice treated with mini DC was also found to be the highest among all groups. In contrast, the increase of effector T cells in BMDC‐treated mice was much modest (Figure 5F; Figure S9A, Supporting Information). In addition, there was lower percentage of CD4^+^Foxp3^+^ T_reg_ in the spleens of mini DC–immunized mice in comparison with mice of other groups (Figure 5G; Figure S9B, Supporting Information). Taken together, these data validated the immune stimulation ability of mini DC in tumor‐bearing mice, thus conferring favorable therapeutic antitumor efficacy in an ovarian cancer model.

### 2.5 Suppression of Abdominal Metastasis of Ovarian Cancer by Mini DC

**Figure 6:**
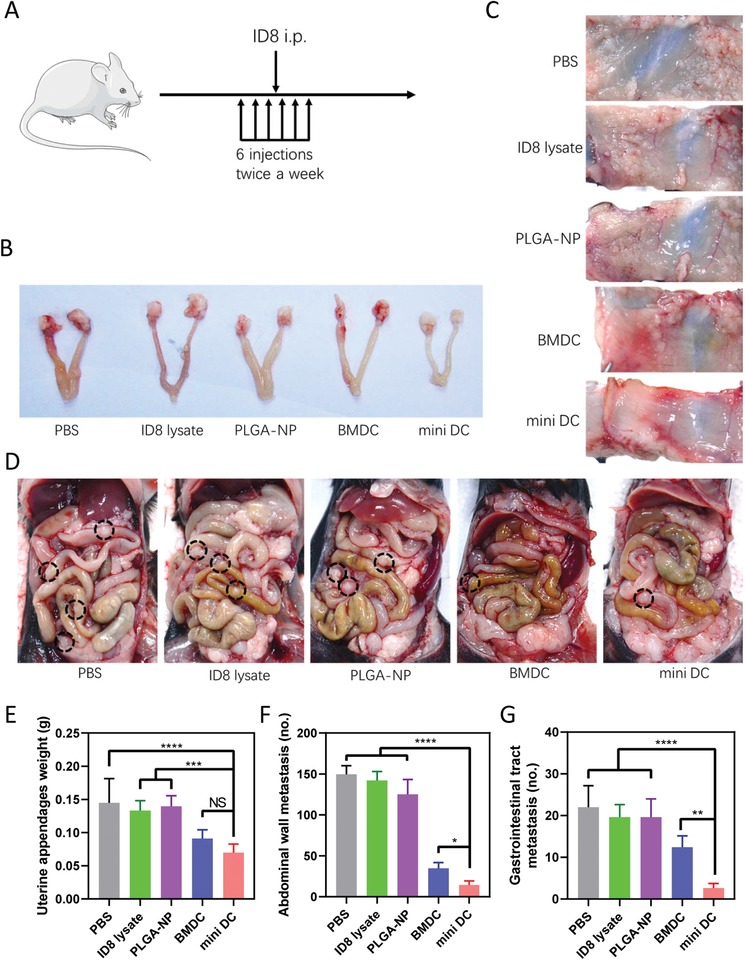
Metastasis inhibition effect of mini DC. A) Schematic diagram for the regimen of antimetastasis immunization of mice on indicated days. After three vaccinations, mice were challenged with ID8 mouse ovarian cancer cells intraperitoneally, followed by another three doses (*n* = 5 biologically independent animals in each group). B) Representative digital images and E) weight of uterine appendages in mice vaccinated with different formulations. Representative digital images and statistical analysis for the number of the metastatic nodules on C,F) abdominal walls and D,G) gastrointestinal tracts from immunized mice 1 month after tumor cell inoculation. Black circles indicated the location of the metastatic nodules. In panels (E)–(G), data are represented as mean ± SD. One‐way ANOVA with Dunnett's posthoc analysis was used for the comparison between groups. ****p < 0.0001, ***p < 0.001, **p < 0.01, and *p < 0.05. NS: no significance.

Given the fact that advanced ovarian cancer is characterized by widespread abdominal dissemination, we next examined the effectiveness of mini DC in inhibition of ovarian cancer metastasis. Grouped female C57BL/6 mice were first vaccinated with three doses of different vaccine formulations, followed by intraperitoneal implantation of ID8 cells and another three doses of formulations (**Figure** **6**A). One month after tumor cell inoculation, mice were sacrificed to inspect metastatic tumor nodules in the abdominal cavity. Evident increase of the weight of uterine appendages due to cancer cell colonization was observed in mice of PBS, ID8 lysate, and PLGA‐NP groups, while the counterparts in BMDC or mini DC–treated mice were relatively smaller (Figure 6B,E). Impressively, the number of metastatic tumor nodules on the abdominal wall of mini DC–vaccinated mice was tenfold lower than that of the PBS control group and less than half of the number in the BMDC group (Figure 6C,F). Similar result was also observed in gastrointestinal tract, where minimal nodules could be found in the mini DC group (Figure 6D,G). These findings verified the potential of mini DC in suppression of ovarian cancer metastasis.

### 2.6 Biocompatibility and Safety Assessment of Mini DC

**Figure 7:**
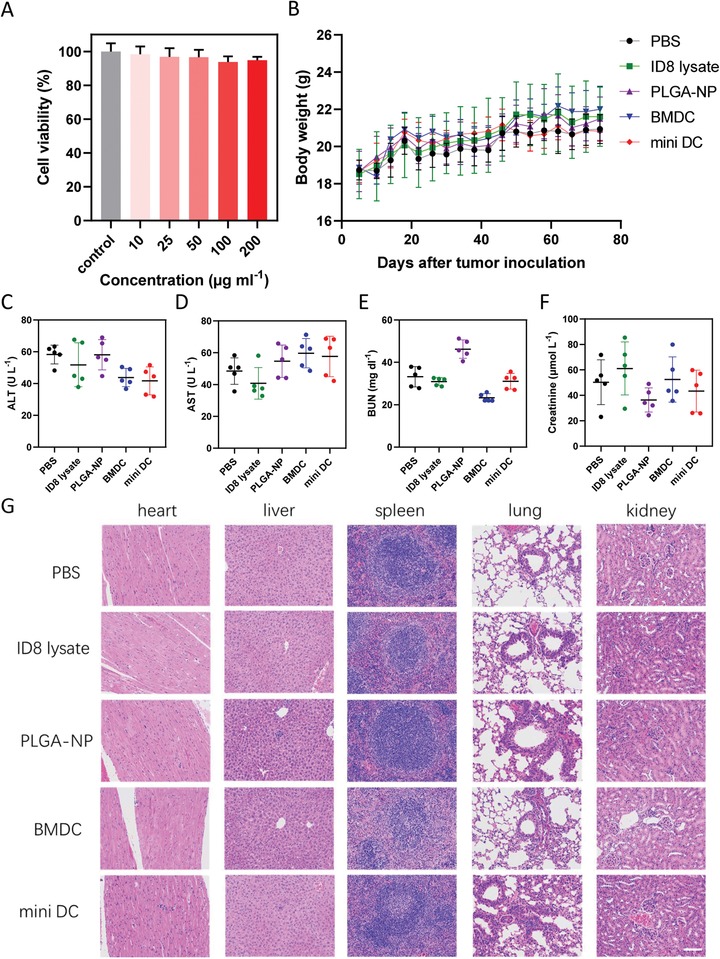
Safety evaluation of mini DC. A) Cell viability of NIH‐3T3 cells incubated with various concentrations of mini DC for 24 h measured by CCK‐8 (*n* = 3). B) Body weight of the tumor‐bearing mice following tumor inoculation s.c. and corresponding treatments. C) ALT, D) AST, E) BUN, and F) creatinine levels in the serum of tumor‐bearing mice at the end of therapeutic study. G) Representative H&E staining images of heart, liver, spleen, lung, and kidney from mice treated with different vaccines. Scale bar: 100 µm. Results in panels (B)–(F) are expressed as mean ± SD, *n* = 5 biologically independent animals in panels (B)–(G).

As safety of a pharmaceutical is of top importance, the biocompatibility of mini DC was finally evaluated. Theoretically, mini DC should be friendly and nontoxic to organisms as it is synthesized by purely natural cell membrane and biodegradable PLGA, which has been approved by the US FDA in drug delivery systems.^41^ Our data testified this hypothesis both in vitro and in vivo. For the in vitro study, NIH‐3T3 or 293T cells were incubated with various concentrations of mini DC for 24 h, after which Cell Counting Kit‐8 (CCK‐8) was used to determine the viability of cells. As shown in **Figure** **7**A, the viability of cells is kept above 90% without being influenced by the increase of concentration. The same result was obtained in 293T cells (Figure S10A, Supporting Information). Hemolysis assay also verified the in vitro biocompatibility as no significant hemolysis was found in the presence of mini DC even at a concentration as high as 200 µg mL^−1^ (Figure S10B, Supporting Information).

The in vivo biocompatibility of mini DC was assessed mainly through monitoring indices of mice in the therapeutic study. The body weight of mini DC–vaccinated mice remained stable and comparable to that of the mice in other groups during the course of treatment (Figure 7B). The serum hepatic enzymes (alanine aminotransferase (ALT)/aspartate aminotransferase (AST)) and kidney function parameters (blood urea nitrogen (BUN) and creatinine) levels were within the reference values at the end of the study (Figure 7C–F). Moreover, histological analysis with hematoxylin and eosin (H&E)–stained sections of major organs also indicated that no obvious damages were observed, demonstrating good in vivo biocompatibility of mini DC (Figure 7G).

## 3 Conclusion

In summary, we have developed a biomimetic cancer nanovaccine based on DCs for ovarian cancer immunotherapy in this study. This nanovaccine was prepared by encapsulating IL‐2‐loaded PLGA‐NP with the cell membrane derived from tumor cell lysate‐pulsed mature DCs. Inheriting critical membrane proteins from their source cells, this synthetic DC mimic can present antigen and stimulate T cells through the unique interaction between them. Here we demonstrate that it elicited enhanced T‐cell response compared with DC both in vitro and in vivo. This improvement may attribute to the nanosize effect, which helps to break the temporal and spatial limits during the antigen presentation process. Meanwhile, it could also overcome some major shortcomings of DC vaccines, including short shelf‐life and vulnerability to unfavorable immunosuppressive conditions because of its nonliving essence. These advantages were demonstrated by significantly augmented efficacies of tumor growth delay and metastasis suppression in a mouse ovarian cancer model. Overall, the nanovaccine we proposed herein is of high clinical value as a facile strategy for antitumor immunotherapy and personalized vaccination.

## 4 Experimental Section

##### Reagents

β‐mercaptoethanol (catalog: M3148), polyvinyl alcohol (PVA, catalog: SLBV3641), HOCl (catalog: 239 305), Pluronic F‐127 (catalog: P2443), and lactic acid (catalog: L1750) were obtained from Sigma–Aldrich (Saint Louis, USA). Carboxyl‐terminated 50:50 (PLGA, catalog: B6010‐2) was purchased from DURECT Corp. (Birmingham, USA). Bicinchoninic acid (BCA) kit (catalog: P0012S), Coomassie Blue Staining Solution (catalog: ST030), and Mycoplasma Detection Kit (catalog: C0296) were purchased from Beyotime Bioteccnology (Haimen, China). CCK‐8 (catalog: CK04) and calcein‐AM (catalog: C326) were purchased from Dojindo (Kumamoto, Japan). Indocyanine green (catalog: 412 545 000) was purchased from Acros Organics (Belgium, USA). Lipopolysaccharide (LPS, catalog: 00‐4976‐03), (CFSE, catalog: 65‐0850‐84), phenylmethylsulfonyl fluoride (PMSF, catalog: 36 978), Protease and Phosphatase Inhibitor Cocktail (catalog: 78 443), ACK lysis buffer (catalog: 00‐4300‐54), DiD (catalog: D7757), and Hoechst 33 342 (catalog: H1399) were obtained from Thermo Fisher Scientific (MA, USA). Neuro‐DiO (catalog: D4021) was obtained from US Everbright Inc (Jiangsu, China). Murine recombinant granulocyte‐macrophage colony‐stimulating factor (GM‐CSF) (catalog: AF‐315) was purchased from Peprotech (Rocky Hill, USA). CD8^+^ T‐Cell Isolation MACS kit (catalog: 130‐104‐075) was purchased from Miltenyi Biotec (Teterow, German). Antibodies for western blotting were purchased from Cell Signaling Technology (Danvers, USA), and antibodies for flow cytometry were purchased from eBioscience (Thermo Fisher Scientific, MA, USA) unless otherwise specified. All antibodies were used according to the manufacturer's protocols. Fixable Viability Stain 780 (catalog: 564 995) and anti‐IFN‐γ‐fluorescein isothiocyanate (FITC) (catalog: 554 411) were purchased from BD Bioscience (San Jose, USA). Murine IL‐2 (catalog: 575 408) and Mouse IFN‐γ ELISA MAX Deluxe (catalog: 430 804) were purchased from Biolegend (San Diego, USA), and mouse TNF‐alpha Quantikine ELISA kit (catalog: MTA00B) was purchased from R&D systems (Minneapolis, USA). Mouse IL‐2 ELISA kit (catalog: 70‐EK202) was purchased from MultiSciences Biotech (Hangzhou, China). All cell culture reagents including Roswell Park Memorial Institute (RPMI) 1640 (catalog: A1049101), Dulbecco's modified Eagle medium (DMEM) (catalog: 11 965 092), fetal bovine serum (FBS, catalog: 10 099 141), penicillin–streptomycin (catalog: 15 140 122), and trypsin–ethylenediaminetetraacetic acid (0.25%, catalog: 25 200 072) were purchased from Gibco (Thermo Fisher Scientific, MA, USA).

##### Cell Culture

NIH‐3T3 and 293T cells were purchased from the Cell Bank of Type Culture Collection of Chinese Academy of Sciences. Murine ovarian cancer cell line ID8 was a gift from the University of Kansas Medical Center. Cell lines were cultured in DMEM medium supplemented with 10% FBS and 1% penicillin–streptomycin at 37 °C in 5% CO_2_ for no more than ten passages. All cell lines were routinely tested for mycoplasma contamination and confirmed to be negative.

##### Animals

C57BL/6 mice were provided by and housed in an animal facility at Renji Hospital under specific pathogen‐free (SPF) condition. All animal procedures were performed in accordance with ethical guidelines and approved by Institutional Animal Care and Use Committee of Shanghai Jiao Tong University School of Medicine (ethic code: A2019114).

##### Preparation of Tumor Cell Lysate

The preparation of whole HOCl‐oxidized tumor cell lysate was modified from a protocol reported in the previous study.^32^, ^33^ ID8 ovarian cancer cells were incubated in PBS containing 60 × 10^−6^ m HOCl at a density of 1 × 10^6^ cells mL^−1^ for 1 h. After confirmation of cells' death, which was assessed by trypan blue staining, cells were collected and washed twice with PBS. Then tumor cells were frozen in liquid nitrogen for 10 min and thawed completely at room temperature for six cycles, after which cells were homogenized using a dounce homogenizer (KIMBLE, Millville, USA) with a loose pestle for 50 strokes to complete tumor cell fragmentation.

##### BMDC Isolation, Differentiation, and Antigen Presentation Assays

DCs were differentiated from bone marrow cells collected from the femurs and tibiae of 8–10 week old male or female C57BL/6 mice. Single‐cell suspensions were cultured in media containing RPMI 1640, 10% FBS, 1% penicillin–streptomycin, 50 × 10^−6^ m β‐mercaptoethanol, 2 × 10^−3^ m l‐glutamine, 20 ng mL^−1^ murine recombinant GM‐CSF. Culture media were replenished on days 3 and 6. To assess the maturation and antigen presentation of DCs pulsed with tumor cell lysate, nonadherent cells between days 7 and 9 were collected and incubated with neuro‐DiO‐labeled tumor cell lysate prepared from ID8 cells with equal number for 36 h. Then DCs were harvested and stained with anti‐CD11c (catalog: 11‐0114‐85), anti‐CD86 (catalog: 17‐0862‐82), and anti‐MHCII (catalog: 12‐5321‐82) for 30 min on ice, washed, and analyzed using flow cytometry (CytoFLEX, Beckman Coulter).

##### Preparation of IL‐2 Loaded PLGA‐NP

The PLGA polymeric cores were prepared using PLGA polymer (50:50) through a double‐emulsion method.^28^ Briefly, 60 µL aqueous IL‐2 solution containing 100 ng IL‐2 was added to 10 mg PLGA in 500 µL dichloromethane drop by drop while vortexing. The mixture was sonicated using a sonicator (SCIENTZ‐IID, Ningbo Scientz Biotechnology Co.) with an amplitude of 30% for a 2 min pulse (2 s on/2 s off), and added dropwise to a continuously vortexed tube with 6 mL water containing 4% PVA. This resulting double emulsion was sonicated at the same setting for 5 min in an ice bath and then transferred to a vacuum aspirator to remove the solvent. Hardened nanoparticles were washed three times by pelleting at 21 000 × *g* for 30 min and resuspension in Milli‐Q water and lyophilized for long‐term storage. For fluorescent label purpose, 20 µg of DiD was added into the PLGA dichloromethane solution prior to the addition of IL‐2 solution.

##### Preparation and Characterization of Mini DC

Derivation of plasma membrane of DCs was adapted from a previous published protocol with minor modification.^42^ Briefly, 5 × 10^7^ mature DCs verified above were collected and resuspended in 2 mL of hypotonic lysing buffer consisting of 25 × 10^−3^ m sucrose, 10 × 10^−3^ m Tris/HCl, 1 × 10^−3^ m MgCl_2_, 1 × 10^−3^ m KCl, 2 × 10^−3^ m PMSF, and 1 × Protease and Phosphatase Inhibitor Cocktail on ice for 30 min. Cells were then enucleated using a hand‐held Dounce homogenizer with a tight‐fitting pestle (25 passes while on ice), followed by centrifuging at 800 × *g* for 5 min at 4 °C. The supernatant was collected and the pellet was suspended in the hypotonic lysing buffer again. The homogenization and centrifugation steps were repeated for another three times to make sure that the pellet was free of intact cells as viewed by light microscopy. The supernatants were pooled and centrifuged again at 21 000 × *g* for 10 min at 4 °C. The enriched membranes were dispersed in PBS on ice and washed twice at 4 °C, after which their contents were quantified by a BCA kit. For cell membrane coating, DC membranes were mixed with PLGA‐NP at a membrane protein‐to‐nanoparticle weight ratio of 1:1. The mixture was then physically extruded through 1 µm and 400 nm polycarbonate membranes for 11 passes successively using a miniextruder (Avanti, USA). The resulting nanoparticles, namely, mini DC, were visualized through a transmission electron microscope (HT7700, HITACHI, Japan) after being stained with uranyl acetate (1 wt%) to get their morphological information. Hydrodynamic size and surface zeta potential of mini DC were measured with zetasizer (Nano‐ZS, Malvern) for further confirmation of successful cell membrane coating. The total protein contents of BMDC lysate, BMDC membrane–derived vesicle (BMDC vesicle), and mini DC were examined by sodium dodecyl sulfate polyacrylamide gel (SDS‐PAGE) electrophoresis and imaged by Coomassie blue staining. Specific surface markers on BMDC, BMDC vesicle, and mini DC were examined with western blotting. After being loaded with proteins extracted from them, polyvinyl difluoride (PVDF) membrane was probed using antibodies against mouse CD11c, CD86, and CD40. Based on quantification of protein band of CD11c, ≈2 million BMDCs were able to yield mini DC with a protein weight of 0.25 mg. To assess the orientation of proteins on mini DC, BMDC and mini DC with equivalent membrane proteins were stained with PE‐labeled MHC II antibody for 30 min on ice, washed, and the fluorescence intensity was measured through a microplate reader (Synergy H4, Biotek). To examine cytokine release, 1 mg of PLGA‐NP and mini DC prepared from equivalent PLGA‐NP were incubated in 1 mL of PBS containing 1% Pluronic F‐127 at 37 °C with continuous shaking. IL‐2 concentrations of supernatant samples collected from various time points were measured using an ELISA kit.

##### Mini DC Adhesion Assay

To evaluate the specific targeting of nanoDCs to T cells, primary CD8^+^ T cells were isolated from mouse spleens using CD8^+^ T‐Cell Isolation MACS Kit according to manufacturer's protocol. DiD‐labeled PLGA‐NPs and nanoDCs were incubated with CD8^+^ T cells or NIH‐3T3 cells with a concentration of 0.2 mg mL^−1^ at 4 °C for 1 h. Then cells were fixed and stained using Neuro‐DiO and Hoechst 33 342, washed, and observed under confocal laser scanning microscopy (Leica TSC SP8). Flow cytometry was also used for analysis of targeting efficiency of nanoDCs to CD8^+^ T cells.

##### In Vitro T‐Cell Activation and Proliferation Study

CD8^+^ T cells were seeded onto a 96‐well cell culture plate at a density of 2 × 10^5^ per well, and then 50 µL PBS or PBS containing 2 × 10^4^ BMDCs, mini DC with equivalent membrane protein content, ID8 lysate prepared from 2 × 10^4^ ID8 cells, or PLGA‐NP (equivalent with mini DC as quantified via fluorescence) were cocultured with T cells at 37 °C. After 24 h co‐culture, T cells were collected and stained with anti‐CD8‐PE (eBioscience, catalog:12‐0081‐82) and anti‐CD69‐APC (Biolegend, catalog: 104 513), followed by flow cytometry analysis. For T‐cell proliferation assay, CD8^+^ T cells were stained with CFSE and co‐cultured with formulations described above for 3 days. The culture supernatants were collected for measuring the concentrations of secreted IFN‐γ and TNF‐α by ELISA according to manufacturer's instruction, while T cells were harvested and analyzed by flow cytometry. CFSE dilution was measured to assess the proliferation of T cells.

##### In Vivo Immunization with Mini DC

For the detection of in vivo T‐cell response elicited by mini DC, groups of 6–8 week old female C57BL/6 mice (*n* = 5 for each group) were vaccinated with 100 µL PBS alone or PBS containing ID8 lysate prepared from 2 × 10^6^ ID8 cells, equivalent ID8 lysate‐pulsed BMDC (2 × 10^6^), mini DC with equivalent membrane protein content, or PLGA‐NP (equivalent with mini DC as quantified via fluorescence) subcutaneously at tail base twice a week for 3 weeks. Three days post the last vaccination, peripheral blood of the mice was collected to measure the levels of IFN‐γ and TNF‐α in serum by ELISA. The inguinal lymph nodes and spleens were harvested and dissociated into single cell suspension to test the frequency of total T cells and T_eff_ and T_reg_ using flow cytometry.

##### Ex Vivo Cytotoxicity Assay

To verify whether the T‐cell response is tumor antigen specific, CD8^+^ T cells were further isolated from splenocytes with CD8^+^ T‐Cell Isolation MACS Kit. Specific cytotoxicity of these effector T cells was assessed using calcein‐AM retention assay adopted from a protocol reported before.^43^ Briefly, live ID8 target cells were seeded onto a U‐bottomed 96‐well plate at a density of 3 × 10^4^ cells per well and labeled with 0.02 µg mL^−1^ calcein‐AM in serum and phenol red‐free medium for 30 min at 37 °C. Then CD8^+^ T cells were added at ratios of 20:1 and 10:1 (effector/target) in quadruplicate. Phenol red‐free medium and lysis buffer (1% Trixton X‐100) were added into two 4‐well set of target cells, respectively, to determine retention in medium and retention maximal lysis. After 10 h of incubation, the plate was washed twice and the remaining fluorescence was read. The specific cytotoxicity of effector T cells was calculated as follows: % specific lysis = [(retention experimental well – retention maximal lysis)/(retention in medium – retention maximal lysis)] × 100.

##### Therapeutic and Antimetastasis Studies

For therapeutic study, 6–8 week old female C57BL/6 mice (*n* = 5 for each group) were inoculated with 5 × 10^6^ ID8 ovarian cancer cells subcutaneously at the right flank. One month after tumor inoculation, mice were immunized with different vaccine formulations mentioned above twice a week for 3 weeks. Tumor area and body weight were measured every 4 days, and the tumor area was calculated as length × width. When the tumor area of the PBS control group exceeded 200 mm^2^, all mice were sacrificed and peripheral blood was collected to measure the levels of serum hepatic enzymes and kidney function parameters. The spleens were excised for analysis of the T‐cell subsets by flow cytometry. Tumor masses were excised and sectioned for TUNEL staining and immunofluorescence staining of CTLs. Major organs including heart, liver, spleen, lung, and kidney were collected and examined by H&E staining.

As for antimetastasis study, groups of 6–8 week old female C57BL/6 mice (*n* = 5 for each group) were first immunized with three doses of various vaccine formulations twice a week, after which each mouse was inoculated with 5 × 10^6^ ID8 cells intraperitoneally. Another three doses of vaccines were injected at a same administration frequency. One month after tumor cell inoculation, all mice were sacrificed and abdominal metastatic tumor nodules were then manually counted.

##### Flow Cytometry

The maturation of DCs was examined by staining with fluorescence‐conjugated antibodies including anti‐CD11c‐FITC (eBioscience, catalog: 11‐0114‐85), anti‐CD86‐APC (eBioscience, catalog: 17‐0862‐82), and anti‐MHC Class II‐PE (eBioscience, catalog: 12‐5321‐82). To analyze the T‐cell subsets in spleens and draining lymph nodes of the immunized mice, single cell suspensions prepared from these samples were examined by flow cytometry. The following primary antibodies were used: anti‐CD3e‐PerCP‐Cyanine5.5 (eBioscience, catalog: 45‐0031‐82), anti‐CD8a‐PE (eBioscience, catalog: 12‐0081‐82), anti‐CD4‐FITC (eBioscience, catalog: 11‐0041‐85), anti‐CD25‐APC (eBioscience, catalog: 17‐0251‐82), anti‐Foxp3‐PE (eBioscience, catalog: 12‐4771‐82), anti‐IFN‐γ‐FITC (BD Bioscience, catalog: 554 411), anti‐TNF‐α‐Alexa Fluor 647 (BD Bioscience, catalog: 557 730), and anti‐CD16/CD32 (eBioscience, catalog: MA5‐18012). Flow data were acquired on a CytoFLEX and analyzed using CytExpert (Beckman Coulter, USA) and FlowJo (TreeStar, USA) softwares.

##### Cell Viability Assay

NIH‐3T3 and 293T cells were seeded onto 96‐well plate and cultured at 37 °C. When the cells reached about 90% confluency, 50 µL of mini DC was added at various concentrations ranging from 10 to 200 µg mL^−1^. After 24 h, 10 µL of CCK‐8 solution was added into each well and the absorbance at 450 nm was measured after 2 h of incubation (Varioskan Flash, Thermo Scientific).

##### Hemolysis Assay

Mouse whole blood was centrifuged at 3000 rpm for 5 min and washed three times with PBS to get pure red blood cells (RBCs). Different amounts of mini DC were added into RBC suspension at final concentrations of 10, 25, 50, 100, and 200 µg mL^−1^ and incubated at 37 °C for 2 h. RBC suspension mixed with pure water served as positive control. The samples were centrifuged at 10 000 × *g* for 5 min and the absorbance of supernatants at 540 nm was measured.

##### Statistical Analysis

All data were presented as means ± standard deviation (SD). Statistical analysis was performed using EXCEL (Microsoft, USA) and Prism 8.0 (GraphPad, USA). Unpaired two‐tailed Student's *t*‐test was used for comparison between two groups. A variance similarity test (*F*‐test) was performed before the *t*‐test. For multiple‐group analysis, a one‐way analysis of variance (ANOVA) with Dunnett's posthoc analysis was performed unless otherwise specified. Differences were considered statistically significant if *p* < 0.05 (*p < 0.05, **p < 0.01, ***p < 0.001, and ****p < 0.0001).

## Conflict of Interest

The authors declare no conflict of interest.

## Supporting information

Supporting Information

## References

[1] R. L. Siegel , K. D. Miller , A. Jemal , Ca‐Cancer J. Clin. 2016, 66, 7. 26742998 10.3322/caac.21332

[2] L. A. Torre , B. Trabert , C. E. DeSantis , K. D. Miller , G. Samimi , C. D. Runowicz , M. M. Gaudet , A. Jemal , R. L. Siegel , Ca‐Cancer J. Clin. 2018, 68, 284. 29809280 10.3322/caac.21456 PMC6621554

[3] R. D. Cress , Y. S. Chen , C. R. Morris , M. Petersen , G. S. Leiserowitz , Obstet. Gynecol. 2015, 126, 491. 26244529 10.1097/AOG.0000000000000981 PMC4545401

[4] A. M. Karst , R. Drapkin , J. Oncol. 2010, 2010, 932371. 19746182 10.1155/2010/932371 PMC2739011

[5] G. C. Stuart , Gynecol. Oncol. 2003, 90, S8. 10.1016/s0090-8258(03)00472-4 13129490

[6] J. A. Ledermann , Ann. Oncol. 2017, 28, viii46. 10.1093/annonc/mdx452 29232475

[7] K. Matsuo , V. K. Bond , M. L. Eno , D. D. Im , N. B. Rosenshein , Int. J. Cancer 2009, 125, 2721. 19530239 10.1002/ijc.24654

[8] K. Matsuo , M. L. Eno , D. D. Im , N. B. Rosenshein , A. K. Sood , Gynecol. Oncol. 2010, 116, 61. 19840886 10.1016/j.ygyno.2009.09.018 PMC4162425

[9] K. Odunsi , Ann. Oncol. 2017, 28, viii1. 10.1093/annonc/mdx444 PMC5834124 29232467

[10] J. Hamanishi , M. Mandai , M. Iwasaki , T. Okazaki , Y. Tanaka , K. Yamaguchi , T. Higuchi , H. Yagi , K. Takakura , N. Minato , T. Honjo , S. Fujii , Proc. Natl. Acad. Sci. USA 2007, 104, 3360. 17360651 10.1073/pnas.0611533104 PMC1805580

[11] L. E. Kandalaft , D. J. Powell , N. Singh , G. Coukos , J. Clin. Oncol. 2011, 29, 925. 21079136 10.1200/JCO.2009.27.2369 PMC3068064

[12] A. Gardner , B. Ruffell , Trends Immunol. 2016, 37, 855. 27793569 10.1016/j.it.2016.09.006 PMC5135568

[13] P. W. Kantoff , C. S. Higano , N. D. Shore , E. R. Berger , E. J. Small , D. F. Penson , C. H. Redfern , A. C. Ferrari , R. Dreicer , R. B. Sims , Y. Xu , M. W. Frohlich , P. F. Schellhammer , N. Engl. J. Med. 2010, 363, 411. 20818862 10.1056/NEJMoa1001294

[14] K. F. Bol , G. Schreibelt , W. R. Gerritsen , I. J. de Vries , C. G. Figdor , Clin. Cancer Res. 2016, 22, 1897. 27084743 10.1158/1078-0432.CCR-15-1399

[15] R. L. Sabado , S. Balan , N. Bhardwaj , Cell Res. 2017, 27, 74. 28025976 10.1038/cr.2016.157 PMC5223236

[16] J. L. Tanyi , S. Bobisse , E. Ophir , S. Tuyaerts , A. Roberti , R. Genolet , P. Baumgartner , B. J. Stevenson , C. Iseli , D. Dangaj , B. Czerniecki , A. Semilietof , J. Racle , A. Michel , I. Xenarios , C. Chiang , D. S. Monos , D. A. Torigian , H. L. Nisenbaum , O. Michielin , C. H. June , B. L. Levine , D. J. Powell , D. Gfeller , R. Mick , U. Dafni , V. Zoete , A. Harari , G. Coukos , L. E. Kandalaft , Sci. Transl. Med. 2018, 10, eaao5931. 29643231 10.1126/scitranslmed.aao5931

[17] L. C. Morehead , M. J. Cannon , Ann. Res. Hosp. 2018, 2, 8. 30345421 10.21037/arh.2018.08.02 PMC6192055

[18] P. Verdijk , E. H. Aarntzen , W. J. Lesterhuis , A. C. Boullart , E. Kok , M. M. van Rossum , S. Strijk , F. Eijckeler , J. J. Bonenkamp , J. F. Jacobs , W. Blokx , J. H. Vankrieken , I. Joosten , O. C. Boerman , W. J. Oyen , G. Adema , C. J. Punt , C. G. Figdor , I. J. de Vries , Clin. Cancer Res. 2009, 15, 2531. 19318472 10.1158/1078-0432.CCR-08-2729

[19] C. C. Chang , T. Ogino , D. W. Mullins , J. L. Oliver , G. V. Yamshchikov , N. Bandoh , C. L. Slingluff , S. Ferrone , J. Biol. Chem. 2006, 281, 18763. 16648140 10.1074/jbc.M511525200

[20] J. R. Cubillos‐Ruiz , P. C. Silberman , M. R. Rutkowski , S. Chopra , A. Perales‐Puchalt , M. Song , S. Zhang , S. E. Bettigole , D. Gupta , K. Holcomb , L. H. Ellenson , T. Caputo , A. H. Lee , J. R. Conejo‐Garcia , L. H. Glimcher , Cell 2015, 161, 1527. 26073941 10.1016/j.cell.2015.05.025 PMC4580135

[21] E. Gottfried , L. A. Kunz‐Schughart , S. Ebner , W. Mueller‐Klieser , S. Hoves , R. Andreesen , A. Mackensen , M. Kreutz , Blood 2006, 107, 2013. 16278308 10.1182/blood-2005-05-1795

[22] L. A. O'Neill , E. J. Pearce , J. Exp. Med. 2016, 213, 15. 26694970 10.1084/jem.20151570 PMC4710204

[23] C. M. Hu , L. Zhang , S. Aryal , C. Cheung , R. H. Fang , L. Zhang , Proc. Natl. Acad. Sci. USA 2011, 108, 10980. 21690347 10.1073/pnas.1106634108 PMC3131364

[24] C. M. Hu , R. H. Fang , K. C. Wang , B. T. Luk , S. Thamphiwatana , D. Dehaini , P. Nguyen , P. Angsantikul , C. H. Wen , A. V. Kroll , C. Carpenter , M. Ramesh , V. Qu , S. H. Patel , J. Zhu , W. Shi , F. M. Hofman , T. C. Chen , W. Gao , K. Zhang , S. Chien , L. Zhang , Nature 2015, 526, 118. 26374997 10.1038/nature15373 PMC4871317

[25] Q. Hu , W. Sun , C. Qian , C. Wang , H. N. Bomba , Z. Gu , Adv. Mater. 2015, 27, 7043. 26416431 10.1002/adma.201503323 PMC4998740

[26] C. Zhang , L. Zhang , W. Wu , F. Gao , R. Q. Li , W. Song , Z. N. Zhuang , C. J. Liu , X. Z. Zhang , Adv. Mater. 2019, 31, 1901179. 10.1002/adma.201901179 30924234

[27] C. M. Hu , R. H. Fang , J. Copp , B. T. Luk , L. Zhang , Nat. Nanotechnol. 2013, 8, 336. 23584215 10.1038/nnano.2013.54 PMC3648601

[28] M. D. McHugh , J. Park , R. Uhrich , W. Gao , D. A. Horwitz , T. M. Fahmy , Biomaterials 2015, 59, 172. 25974747 10.1016/j.biomaterials.2015.04.003 PMC5997248

[29] R. H. Fang , A. V. Kroll , W. Gao , L. Zhang , Adv. Mater. 2018, 30, 1706759. 10.1002/adma.201706759 PMC5984176 29582476

[30] P. Kleindienst , T. Brocker , J. Immunol. 2003, 170, 2817. 12626531 10.4049/jimmunol.170.6.2817

[31] L. E. Paulis , S. Mandal , M. Kreutz , C. G. Figdor , Curr. Opin. Immunol. 2013, 25, 389. 23571027 10.1016/j.coi.2013.03.001

[32] C. L. Chiang , J. A. Ledermann , A. N. Rad , D. R. Katz , B. M. Chain , Cancer Immunol. Immunother. 2006, 55, 1384. 16463039 10.1007/s00262-006-0127-9 PMC11030995

[33] C. L. Chiang , L. E. Kandalaft , J. Tanyi , A. R. Hagemann , G. T. Motz , N. Svoronos , K. Montone , G. M. Mantia‐Smaldone , L. Smith , H. L. Nisenbaum , B. L. Levine , M. Kalos , B. J. Czerniecki , D. A. Torigian , D. J. Powell , R. Mick , G. Coukos , Clin. Cancer Res. 2013, 19, 4801. 23838316 10.1158/1078-0432.CCR-13-1185 PMC4049094

[34] R. M. Hochmuth , C. A. Evans , H. C. Wiles , J. T. McCown , Science 1983, 220, 101. 6828875 10.1126/science.6828875

[35] Q. Zhang , D. Dehaini , Y. Zhang , J. Zhou , X. Chen , L. Zhang , R. H. Fang , W. Gao , L. Zhang , Nat. Nanotechnol. 2018, 13, 1182. 30177807 10.1038/s41565-018-0254-4

[36] J. J. Moon , H. Suh , A. V. Li , C. F. Ockenhouse , A. Yadava , D. J. Irvine , Proc. Natl. Acad. Sci. USA 2012, 109, 1080. 22247289 10.1073/pnas.1112648109 PMC3268296

[37] J. Azzi , Q. Yin , M. Uehara , S. Ohori , L. Tang , K. Cai , T. Ichimura , M. McGrath , O. Maarouf , E. Kefaloyianni , S. Loughhead , J. Petr , Q. Sun , M. Kwon , S. Tullius , U. H. von Andrian , J. Cheng , R. Abdi , Cell Rep. 2016, 15, 1202. 27134176 10.1016/j.celrep.2016.04.007 PMC4973867

[38] T. S. Lim , A. Mortellaro , C. T. Lim , G. J. Hammerling , P. Ricciardi‐Castagnoli , J. Immunol. 2011, 187, 258. 21622857 10.4049/jimmunol.1100267

[39] D. W. Kowalczyk , A. P. Wlazlo , W. Giles‐Davis , A. R. Kammer , S. Mukhopadhyay , H. C. Ertl , Cancer Gene Ther. 2003, 10, 870. 14712313 10.1038/sj.cgt.7700653

[40] L. L. van der Woude , M. A. J. Gorris , A. Halilovic , C. G. Figdor , I. J. M. de Vries , Trends Cancer 2017, 3, 797. 29120755 10.1016/j.trecan.2017.09.006

[41] R. A. Jain , Biomaterials 2000, 21, 2475. 11055295 10.1016/s0142-9612(00)00115-0

[42] A. Parodi , N. Quattrocchi , A. L. van de Ven , C. Chiappini , M. Evangelopoulos , J. O. Martinez , B. S. Brown , S. Z. Khaled , I. K. Yazdi , M. V. Enzo , L. Isenhart , M. Ferrari , E. Tasciotti , Nat. Nanotechnol. 2013, 8, 61. 23241654 10.1038/nnano.2012.212 PMC3751189

[43] D. Matza , A. Badou , M. K. Jha , T. Willinger , A. Antov , S. Sanjabi , K. S. Kobayashi , V. T. Marchesi , R. A. Flavell , Proc. Natl. Acad. Sci. USA 2009, 106, 9785. 19497879 10.1073/pnas.0902844106 PMC2701053

