# Supplementary material for: Artificial Mini Dendritic Cells Boost T Cell–Based Immunotherapy for Ovarian Cancer
Source: Adv Sci (Weinh). 2020 Feb 5;7(7):1903301. doi: 10.1002/advs.201903301 (PMC7141030; doi:10.1002/advs.201903301)
Supplement: Supplementary file 1 — Supporting Information [file ADVS-7-1903301-s001.pdf]

Copyright WILEY-VCH Verlag GmbH & Co. KGaA, 69469 Weinheim, Germany, 2020.

ADVANCED SCIENCE Open Access

Supporting Information

for *Adv. Sci.,* DOI: 10.1002/advs.201903301

Artificial Mini Dendritic Cells Boost T Cell–Based Immunotherapy for Ovarian Cancer

*Shanshan Cheng, Cong Xu, Yue Jin, Yu Li, Cheng Zhong, Jun Ma, Jiani Yang, Nan Zhang, Yuan Li, Chao Wang, Zhiyou Yang, and Yu Wang**

Copyright WILEY-VCH Verlag GmbH & Co. KGaA, 69469 Weinheim, Germany, 2020.

Supporting Information

Artificial mini-dendritic cell boosts T cell-based immunotherapy for ovarian cancer

*Shanshan Cheng, Cong Xu, Yue Jin, Yu Li, Cheng Zhong, Jun Ma, Jiani Yang, Nan Zhang, Yuan Li, Chao Wang, Zhiyou Yang, Yu Wang**

*Corresponding author:
E-mail: renjiwangyu@126.com

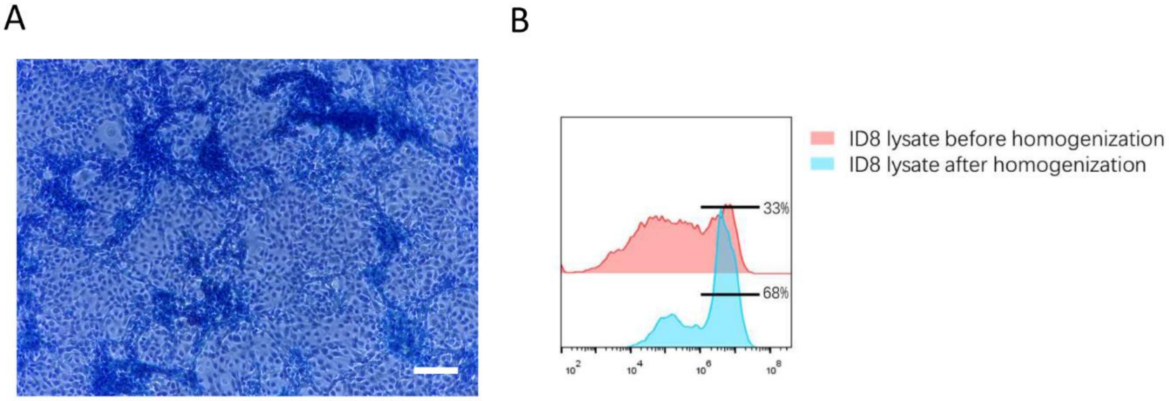

Figure S1. A) Representative image of HOCl-oxidized ID8 cells after trypan blue staining. Scale bar: 100 µm. B) Representative FACS histogram of Neuro-DiO-positive murine BMDC after stimulation with Neuro-DiO-labeled ID8 lysate processed by homogenization (blue) or not (red).

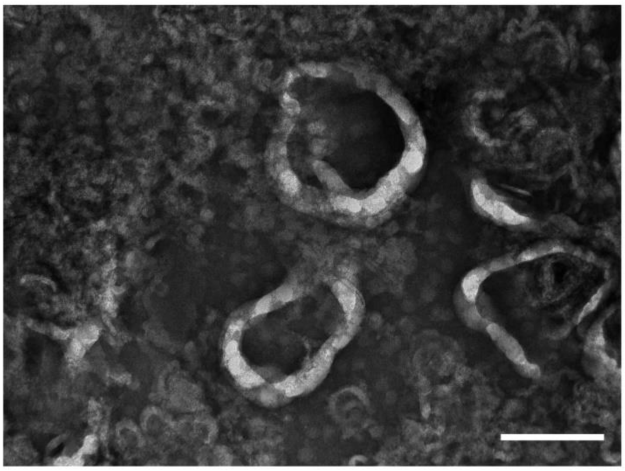

Figure S2. Representative TEM image of BMDC vesicle stained with uranyl acetate. Scale bar: 200 nm.

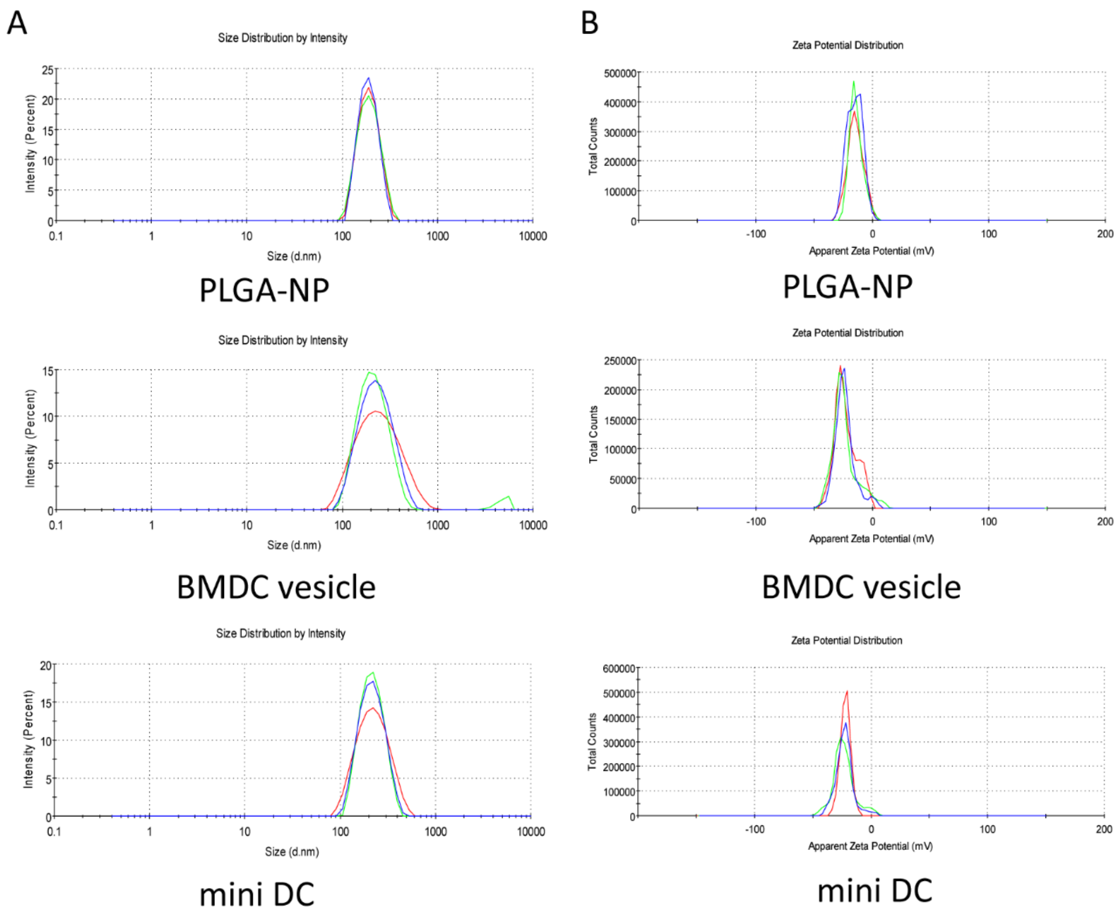

Figure S3. Representative curves of A) hydrodynamic size and B) zeta potential of PLGA-NP, BMDC vesicle and mini DC measured by DLS. Curves with different colors ((green, red and blue) represent three independent tests for the one sample in each subfigure.

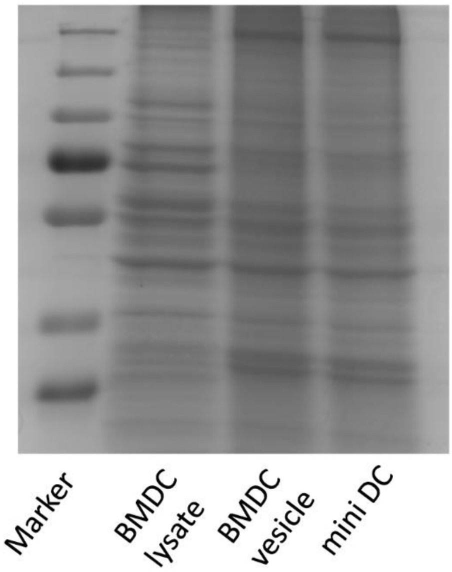

Figure S4. SDS-PAGE protein analysis of BMDC lysate, BMDC vesicle and mini DC, respectively.

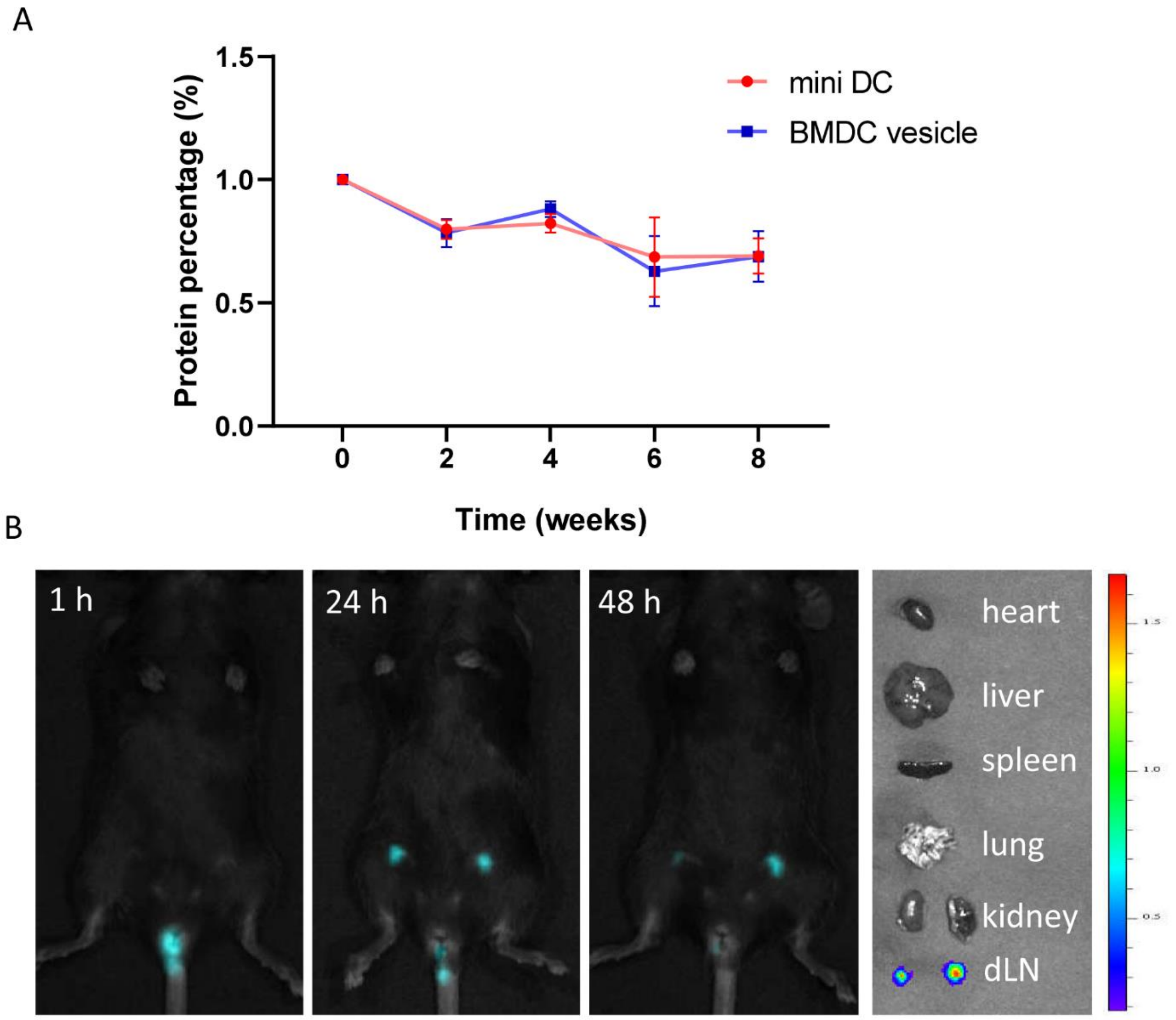

Figure S5. A)Storage stability of mini DC evaluated by content of total proteins over 8 weeks stored at -20℃. B) *In vivo* NIR fluorescence imaging of C57BL/6L mice (n=3) 1 h, 24 h and 48 h after subcutaneous immunization with ICG labeled mini DC at the base of tail. After 48 h, inguinal lymph nodes and major organs were collected, and ex vivo imaging showed high mini DC accumulation in the lymph nodes over other organs.

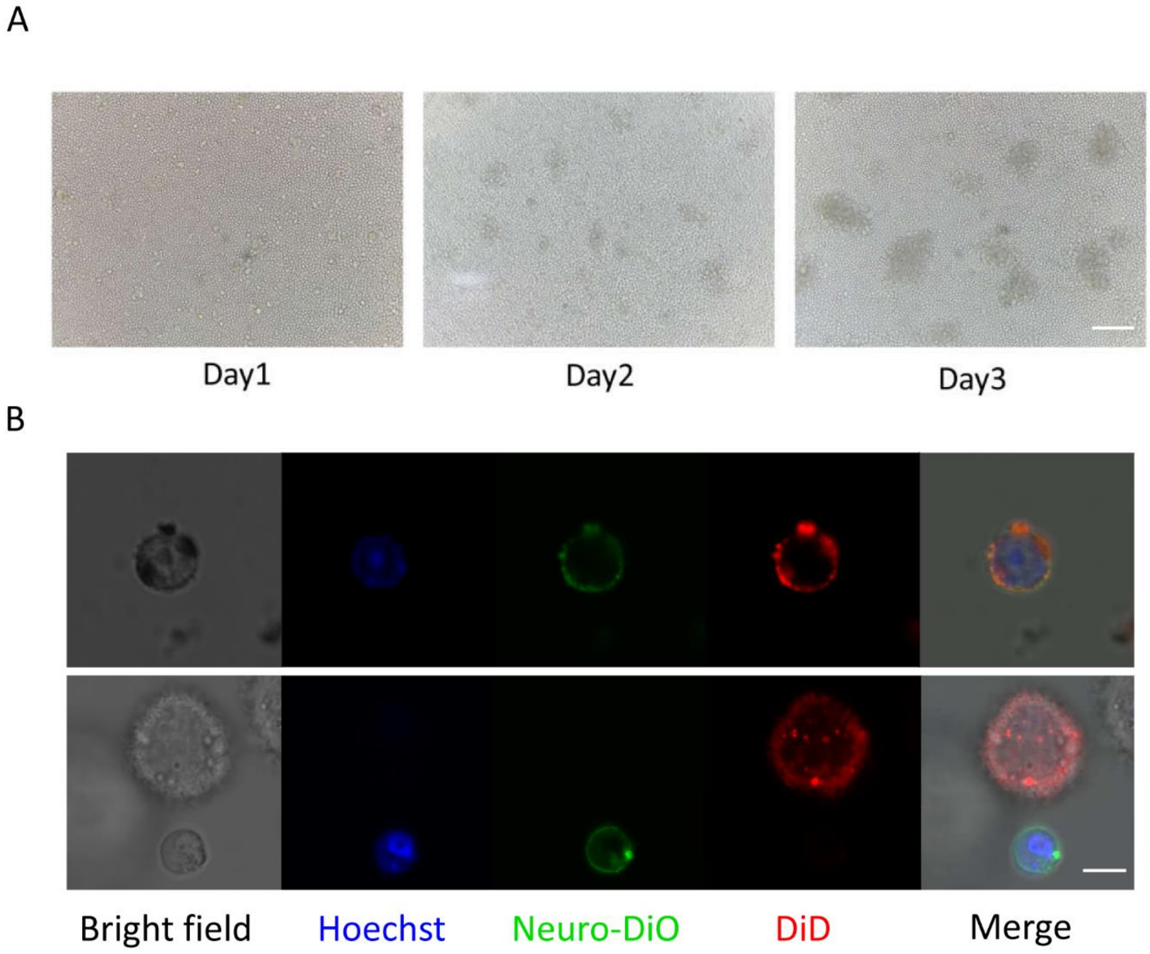

Figure S6. A)Representative images of primary CD8+ T cells incubated with mini DC for 1, 2 and 3 days. Scale bar: 100 μm. B) Comparison between the interaction of mini DC with T cell (top) and the interaction of BMDC with T cell (bottom). Both mini DC and BMDC were labeled with DiD (red) and T cell was labeled with Neuro-DiO (green). Scale bar: 10 μm.

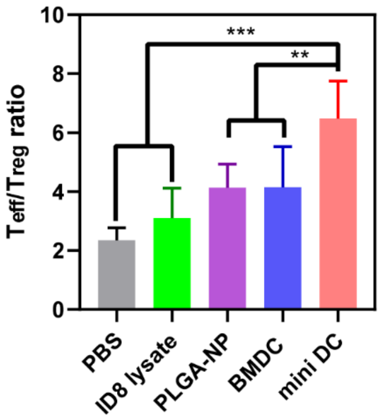

Figure S7. The ratio of T_{eff} ($CD3^+CD8^+$)/T_{reg} ($CD4^+CD25^+Foxp3^+$) in the spleens of immunized mice in different groups. Data showed mean ± SD and were analyzed by one-way ANOVA with Dunnett's post hoc analysis, **P < 0.01, ***P < 0.001.

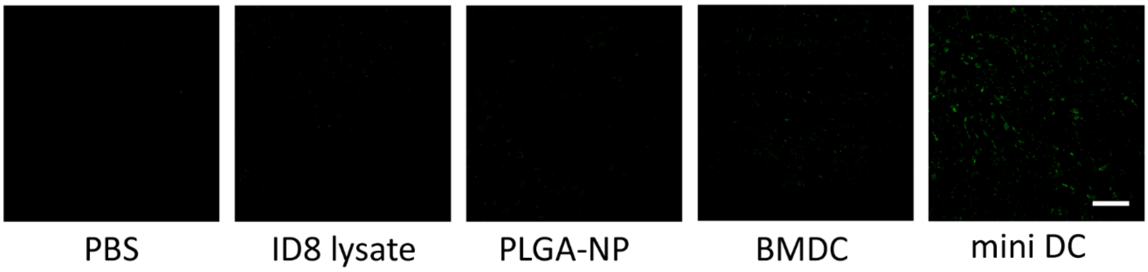

Figure S8. Representative TUNEL staining images of tumor masses harvested from mice vaccinated with different formulations. Scale bar: 50 μm.

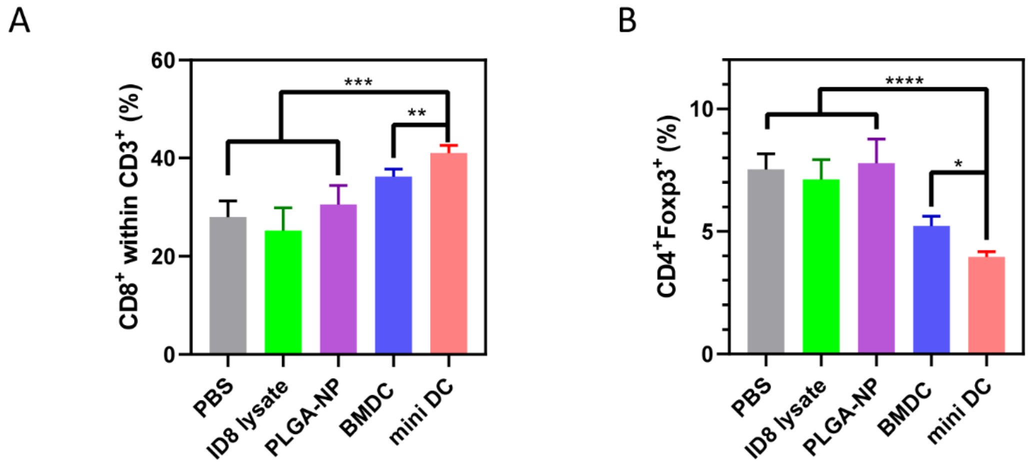

Figure S9. Percentage of A) $CD8^{+}CD3^{+}$ T cells and B) $CD4^{+}Foxp3^{+}$ T cells in the spleens of tumor-bearing mice receiving different treatments. Data depicted mean ± SD. and were analyzed by one-way ANOVA with Dunnett's post hoc analysis, *P < 0.05, **P < 0.01, ***P < 0.001.

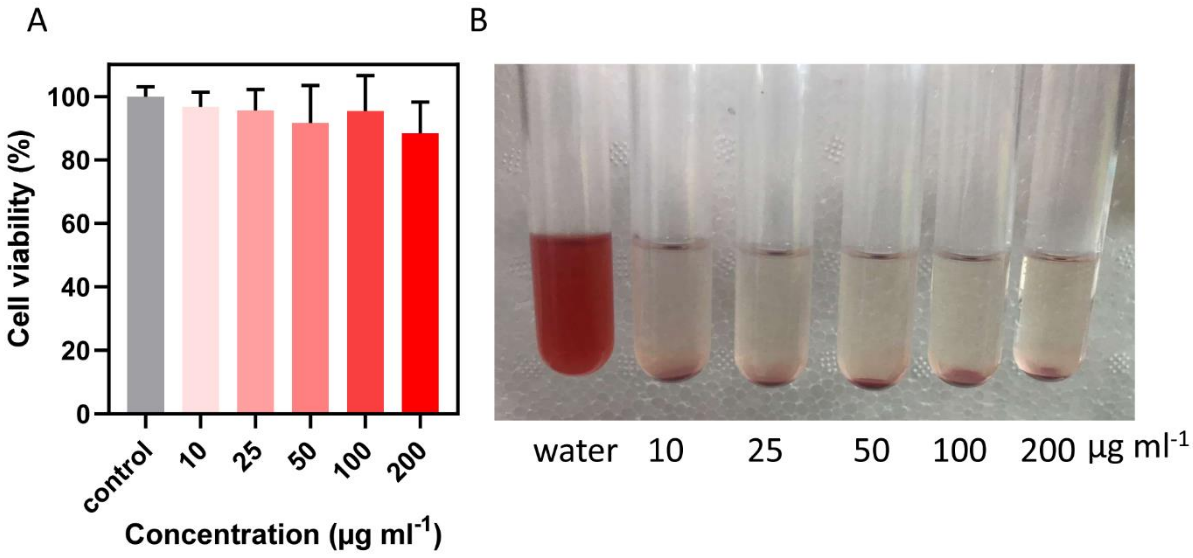

Figure S10. A) Cell viability of 293T cells incubated with various concentrations of mini DC for 24 hours measured by CCK-8 (n=3). B) Representative image of RBCs incubated with mini DC at different final concentrations for 2 hours.
